# Meta-analysis of efficacy and safety of bevacizumab in the treatment of hereditary hemorrhagic telangiectasia epistaxis

**DOI:** 10.3389/fphar.2023.1089847

**Published:** 2023-12-12

**Authors:** Huimin Chen, Zhiping Zhang, Xiaojuan Chen, Chaoyu Wang, Mingdi Chen, Huizhao Liao, Jinru Zhu, Zhenzhen Zheng, Riken Chen

**Affiliations:** ^1^ The Second Affiliated Hospital of Guangdong Medical University, Zhanjiang, Guangdong, China; ^2^ The People’s Hospital of JiangMen, Jiangmen Hospital, Southern Medical University, Jiangmen, China; ^3^ Sun Yat-Sen Memorial Hospital, Sun Yat-Sen University, Guangzhou, Guangdong, China; ^4^ Taishan Hospital of Traditional Chinese Medicine, Jiangmen, Guangdong, China; ^5^ State Key Laboratory of Respiratory Disease, National Clinical Research Center for Respiratory Disease, Guangzhou Institute of Respiratory Health, The First Affiliated Hospital of Guangzhou Medical University, Guangzhou, Guangdong, China

**Keywords:** bevacizumab, hereditary hemorrhagic telangiectasia epistaxis, treatment, efficacy, meta-analysis

## Abstract

**Objective:** A meta-analysis is conducted to evaluate the effectiveness and safety of bevacizumab in hereditary hemorrhagic telangiectasia (HHT) epistaxis.

**Method:** Two researchers search PubMed, EMBASE and Web of Science databases from their inception until September 3th, 2023. The literature is read and screened, and valid data extracted, collated and analyzed. Its quality is then assessed using the Cochrane risk assessment scale. This study uses Endnote 9.3 software for literature management and RevMan 5.3.1 software for evaluation.

**Results:** A total of 7 documents met the requirements, including a total of 359 patients, and the literature quality evaluation was grade B. The Meta-analysis results showed that:Bevacizumab reduces the Epistaxis Severity Score (ESS) in patients with HHT epistaxis compared with the control [WMD = −0.22,95%CI (−0.38, −0.05), *p* = 0.01]. However, there is no significant effect on duration of epistaxis [WMD = −15.59, 95%CI (−70.41,39.23), *p* = 0.58] and number of epistaxes [WMD = −1.27,95%CI (−10.23,7.70), *p* = 0.78] in patients with HHT epistaxis. In terms of adverse effects, there is no significant difference between the bevacizumab group and control group [OR = 1.36, 95% CI (0.54, 3.44), *p* = 0.52].

**Conclusion:** Bevacizumab is superior to the control group in the treatment of HHT epistaxis, and adverse reactions are not further increased in the bevacizumab group than in the control group, suggesting that bevacizumab has clinical value in the treatment of HHT epistaxis.

## 1 Introduction

Hereditary hemorrhagic telangiectasia (HHT) or Osler-Weber-Rendu syndrome is an autosomal-dominant inherited vascular disease (Fuchizaki et al., 2003) which occurs due to mutations in protein-coding genes that mediate salivary secretion through the transforming growth factor-β (TGF-β) superfamily (Kritharis et al., 2018). The vast majority of HHT epistaxis patients have mutations in endorphilin (ENG) or activin receptor-like kinase 1 (ACVRL1/ALK1), resulting in dysangiogenesis, telangiectasia on the mucosal surface, local hyperfibrinolysis in telangiectasia and arteriovenous malformation in the internal organs (Kwaan and Silverman, 1973; Watanabe et al., 1985; Shovlin, 2010). It is characterized by vascular malformations in the nasal mucosa, skin, gastrointestinal tract, brain, lungs and liver, (Guttmacher et al., 1995) and causes telangiectasia of the nasal mucosa with varying degrees of recurrent epistaxis that can occur in 95% of HHT patients, wherein the mean age of first onset is 12 years and the frequency of epistaxis is approximately 18/month (GRIGG et al., 2017). Severe recurrent epistaxis may last several hours a day, causing severe iron deficiency anemia and often transfusion dependence, and even causing social isolation, as well adversely affecting the patients’ employment, travel and daily activities. Clinical treatments for HHT-related epistaxis mainly include laser, argon plasma coagulation, sclerotherapy, septoplasty of nasal lesions, bevacizumab injection and interventional embolization of epistaxis (Richmon et al., 2007; Harvey et al., 2008). At present, there is no effective treatment for HHT. The bleeding of HHT patients is mostly refractory, and the clinical treatment is still mainly supportive treatment and symptom relief. With the further deepening of research on the pathogenesis of vascular malformation in HHT, newly developed drugs such as bevacizumab (an anti-vascular endothelial growth factor monoclonal antibody) have been used in clinical practice, and bevacizumab has been proven to effectively correct the angiogenesis defects (Li et al., 2018). Studies have shown that bevacizumab is effective in the treatment of familial refractory epistaxis caused by HHT with a high safety profile (Mitchell et al., 2008; Al-Samkari et al., 2019; Vázquez et al., 2020). However, there is currently a lack of multicenter and large-sample randomized controlled trials, and there is little published evidence-based medical literature worldwide. This study aims to conduct a meta-analysis of existing clinical studies to explore the efficacy and safety of bevacizumab in the treatment of HHT epistaxis, thereby providing medical evidence for the use of bevacizumab in patients with HHT epistaxis.

## 2 Data and methods

### 2.1 Literature search

PubMed, Embase and Web of science databases were searched for the following terms: “hereditary hemorrhagic telangiectasia”, “Osler-Weber-Rendu disease”, “epistaxis” and “bevacizumab”. Literature references were traced, the search language was limited to English and the search time was limited to September 3th, 2023.

### 2.2 Inclusion criteria

① Study type: case-control study; ② study subjects: patients with HHT epistaxis; ③ intervention: bevacizumab treatment; ④ outcome indicators: effectiveness indicators (e.g., duration of epistaxis, Epistaxis Severity Score (ESS) and number of epistaxes) and adverse reactions(all treatment-related adverse reactions, such as headache, nausea and vomiting, musculoskeletal pain, edema, dizziness, etc.); ⑤ language: English.

### 2.3 Exclusion criteria

① Abstract, review, or case report; ② duplicate data; ③ does not include test indicators to be evaluated; ④ treatment that affects the efficacy of bevacizumab has been carried out; ⑤ grade C quality standard; ⑥ animal experiments.

### 2.4 Literature screening and data extraction

The titles, abstracts and main texts were independently examined by two researchers to select eligible studies. Selected articles were then reviewed at the full-text level by the same two researchers. Both researchers then independently extracted data from eligible original articles. The main data extracted was author, year, country, study type, sample size, duration of epistaxis, number of epistaxes, ESS, etc. Disagreements regarding literature selection and data extraction were resolved by discussion with a third author.

### 2.5 Literature quality evaluation

The two researchers rated on the Cochrane risk assessment scale as follows: ① random sequence generation; ② allocation concealment; ③ blindness (patients, staff); ④ outcome assessors blinded; ⑤ outcome data completeness; ⑥ selective report outcomes; ⑦ other bias. A “low” (low risk of bias), “high” (high risk of bias) or “unclear” (uncertain) judgment was required for each item. When the evaluation results were inconsistent, a discussion was held with a third author. According to the library RCT article evaluation system, the articles were ultimately graded as A, B or C.

### 2.6 Statistical analysis

Data was analyzed with RevMan 5.3.1 software. The heterogeneity test was conducted first. If the heterogeneity among the studies was not statistically significant (I^2^ < 50% and *p* > 0.1), a fixed effects model was used, and in case of significant heterogeneity (I^2^ > 50% and *p* < 0.1), a random effects model was used. The continuous variables were analyzed by weighted mean difference (WMD) and its 95% confidence interval (CI). The odds ratio (OR) or risk ratio (RR) and its 95% CI were used to analyze the binary variables.

## 3 Results

### 3.1 Literature search results

**FIGURE 1:**
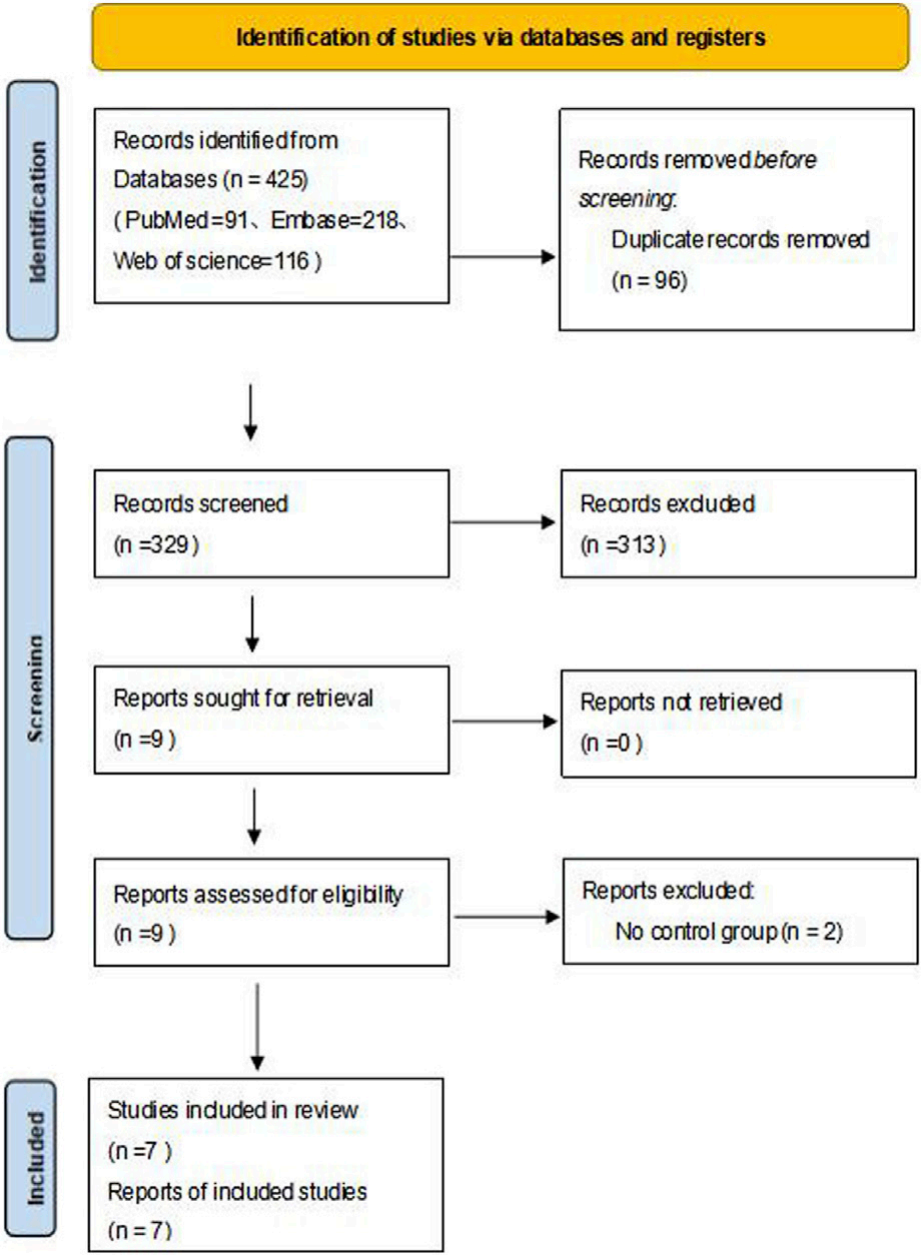
Flow chart of literature screening.

A total of 425 relevant articles were obtained after a preliminary search, and 96 remained after duplicate articles were excluded. After the preliminary screening of titles and abstracts, a total of nine articles met the requirements after excluding case reports, reviews, letters, abstracts, guidelines and articles not belonging to the research field. After reading the full texts, seven articles met the inclusion and exclusion criteria (Simonds et al., 2009; Dupuis-Girod et al., 2014; Riss et al., 2015; Dupuis-Girod et al., 2016; Whitehead et al., 2016; Khanwalkar et al., 2022; Dupuis-Girod et al., 2023). The literature selection flow chart is shown in Figure 1.

### 3.2 Basic characteristics of included studies

Of the seven included studies, the total number of cases was 359. The basic characteristics of all included literature is shown in Table 1.

**TABLE 1:** Basic characteristics of included literature.

Author	Year	Type of study	Sample size	Country	Bevacizumab group	Control group	Outcome indicators
Whitehead et al. (2016)	2016	Randomized controlled trial	120	America	Bevacizumab nasal spray 28 mg	0.9% Normal Saline	①+②+③+④
Dupuis-Girod et al. (2014)	2014	Randomized controlled trial	40	France	Bevacizumab nasal spray 100 mg	0.9% Normal Saline	①+③
Dupuis-Girod et al. (2016)	2016	Randomized controlled trial	80	France	Bevacizumab nasal spray 75 mg	0.9% Normal Saline	①
Simonds et al. (2009)	2009	Retrospective review	19	America	Bevacizumab injection 100 mg + potassium titanylphosphate laser cautery	Potassium titanylphosphate Laser cautery	④
Riss et al. (2015)	2014	Randomized controlled trial	37	Austria	Bevacizumab injection 100 mg	0.9% Normal Saline	①+②+④
Khanwalkar et al. (2022)	2022	Randomized controlled trial	39	America	Bevacizumab injection 25 mg	0.9% Normal Saline	②
Dupuis-Girod S	2023	Randomized controlled trial	24	France	Bevacizumab injection 25 mg	0.9% Normal Saline	①+②+③+④

Note: ① duration of epistaxis; ② Epistaxis Severity Score; ③ number of epistaxis; ④ adverse reactions.

### 3.3 Literature quality evaluation results

**FIGURE 2:**
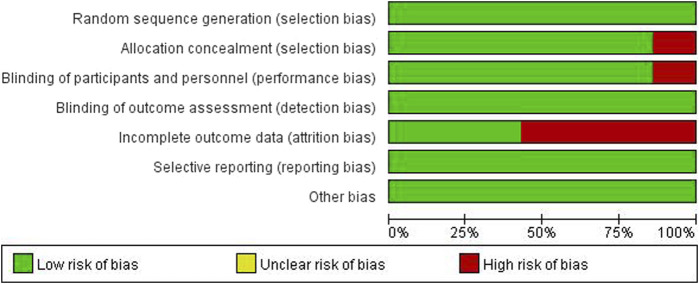
Literature quality evaluation.

**FIGURE 3:**
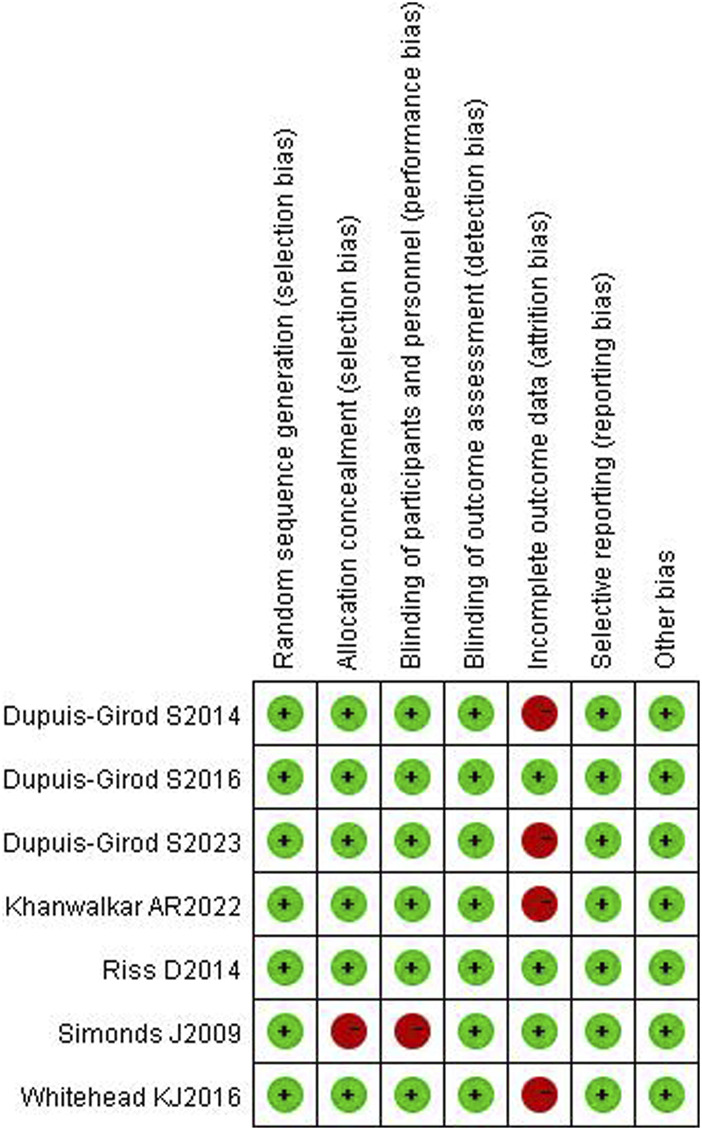
Literature quality evaluation.

Of all included studies, allocation concealment and blindness were not mentioned in the Simonds J (Simonds et al., 2009) study, which was assessed as high risk; in the Whitehead K J (Whitehead et al., 2016) and Khanwalkar AR (Khanwalkar et al., 2022; Dupuis-Girod et al., 2023) studies, case shedding was mentioned, and the completeness of the outcome data was at high risk; the rest were assessed as low risk. All six articles were grade B or above. The results are detailed in Figures 2, 3.

### 3.4 Meta-analysis results

#### 3.4.1 Duration of the epistaxis episode

**FIGURE 4:**
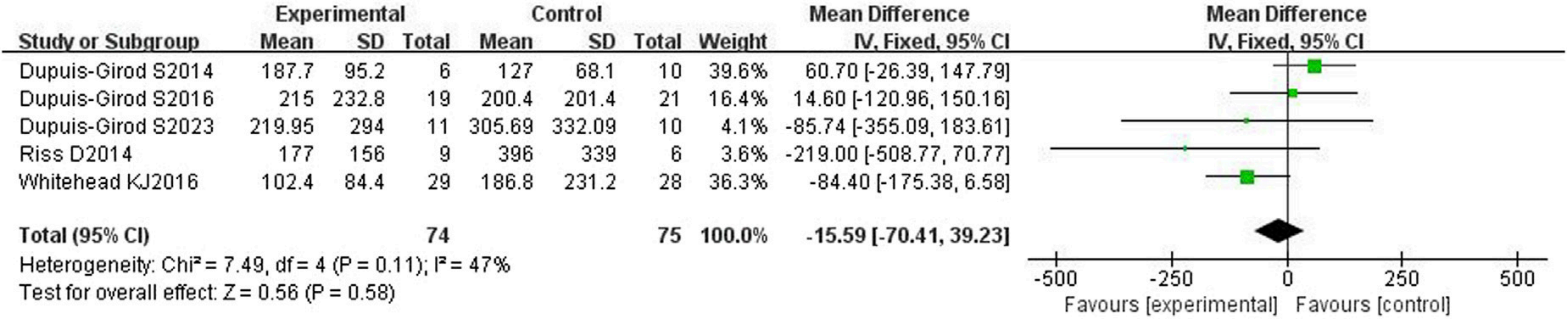
Forest plot of duration of epistaxis in bevacizumab and control groups.

All five included articles (Dupuis-Girod et al., 2014; Riss et al., 2015; Dupuis-Girod et al., 2016; Whitehead et al., 2016; Dupuis-Girod et al., 2023) reported the duration of epistaxis and had a total of 149 patients. The heterogeneity test showed moderate heterogeneity (*p* = 0.11, I^2^ = 47%), so a fixed effects model was used. The results showed that the duration of epistaxis was not significantly different between the bevacizumab group and control group [WMD = −15.59, 95%CI (−70.41, 39.23), *p* = 0.58], as shown in Figure 4. After excluding each study one by one for sensitivity analysis, it was found that the duration of nasal hemorrhage in the bevacizumab group was still not significantly different from that in the control group, and the results were stable.

#### 3.4.2 ESS score

**FIGURE 5:**
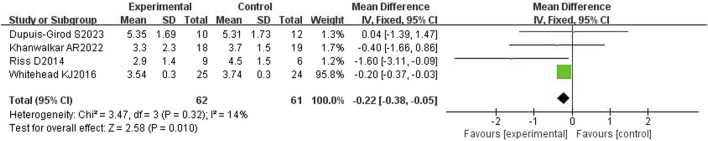
Forest plot of ESS in bevacizumab and control groups.

All four included articles (Riss et al., 2015; Whitehead et al., 2016; Khanwalkar et al., 2022; Dupuis-Girod et al., 2023) reported the ESS and had a total of 131 patients. The heterogeneity test showed mild heterogeneity (*p* = 0.32, I^2^ = 14%), so a fixed effects model was used. The results showed that the ESS in the bevacizumab group was statistically significant compared with the control group [WMD = −0.22,95%CI (−0.38, −0.05), *p* = 0.01], as shown in Figure 5. However, there was no statistical difference between the duration of epistaxis in the bevacizumab group and the ESS score in the control group, which requires further confirmation by large sample and multi-center clinical studies.

#### 3.4.3 Number of epistaxes

**FIGURE 6:**
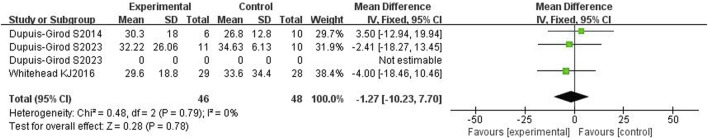
Forest plot of number of epistaxes in bevacizumab and control groups.

All three included articles (Dupuis-Girod et al., 2014; Whitehead et al., 2016; Dupuis-Girod et al., 2023) reported the number of epistaxes and had a total of 94 patients. The heterogeneity test showed no heterogeneity (*p* = 0.79, I^2^ = 0%), so a fixed effects model was used. The results showed that the number of epistaxes in bevacizumab group was not statistically significant compared with the control group [WMD = −1.27,95%CI(−10.23, 7.70), *p* = 0.78], as shown in Figure 6. After removing each study one by one for sensitivity analysis, it was found that there was no significant difference in the number of epistaxis between the bevacizumab group and the control group, and the results were stable.

#### 3.4.4 Adverse reactions

**FIGURE 7:**
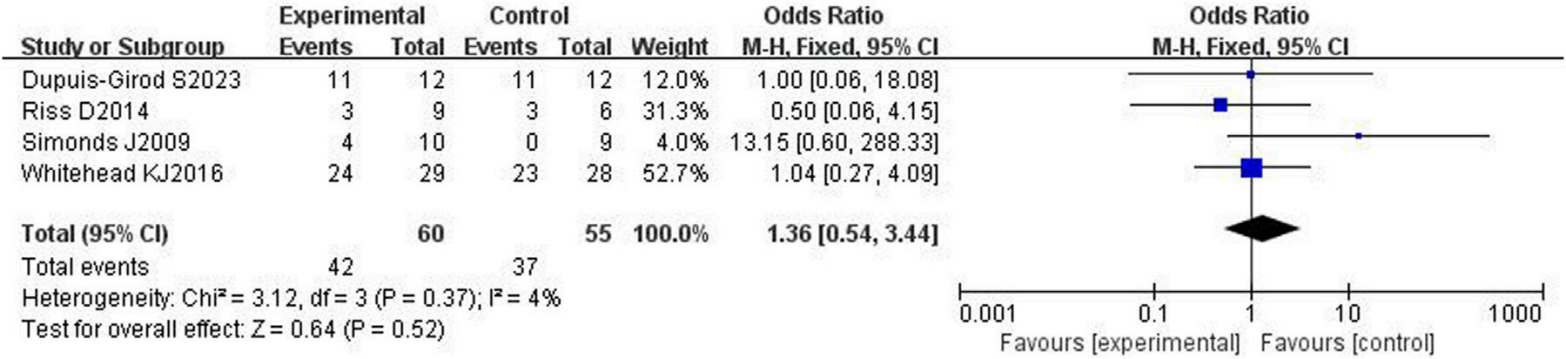
Forest plot of adverse effects in bevacizumab and control groups.

All four included articles^[14, 17–18,20]^ reported adverse effects in a total of 115 patients. The heterogeneity test showed mild heterogeneity (*p* = 0.37, I2 = 4%), so a fixed effects model was used. The results showed no significant difference in adverse effects between the bevacizumab group and control group [OR = 1.36, 95% CI (0.54,3.44), *p* = 0.52], as shown in Figure 7. After removing each study one by one for sensitivity analysis, it was found that there was no significant difference in adverse reactions between the bevacizumab group and the control group, and the results were stable.

### 3.5 Publication bias evaluation

**FIGURE 8:**
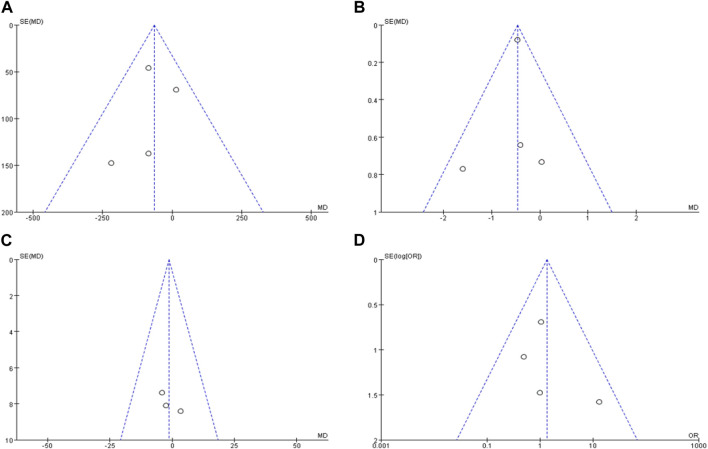
Publication bias funnel plots of included literature. Note: **(A)** is the funnel plot of the duration of the epistaxis episode, **(B)** is the funnel plot of ESS score, **(C)** is the funnel plot number of epistaxes, **(D)** is the funnel plot of adverse reactions.

The publication bias of the included studies was analyzed by mapping the funnel plots. The left and right distributions of each study site were largely symmetrical without severe publication bias, as shown in Figure 8.

## 4 Discussion

Spontaneous, repeated and massive epistaxis is the most obvious feature of HHT, which often leads to chronic and severe anemia, and requires iron supplements and even frequent blood transfusions to keep patients alive. Although electrocoagulation or arterial embolization can immediately relieve epistaxis, their effects are short-lived. HHT can be divided into six types according to different pathogenic genes: HHT1 (ENG gene), HHT2 (ACVRL1 gene), HHT3 and HHT4 (on chromosomes 5 and 7 respectively, with pathogenic gene mapping but not yet cloned), juvenile polyposis and HHT syndrome (SMAD4 gene), and HHT5 (GDF2 gene). Pathologically, the relevant proteins encoded by these genes play a role in the TGF-β and vascular endothelial growth factor (VEGF) signaling cascade, with TGF-β and VEGF concentrations elevated in patient serum and mucosa. Currently, the inhibition of VEGF is a rational way to treat HHT and a pharmacological basis for the application of bevacizumab, a known VEGF inhibitor, in the treatment of the disease (ten Dijke and Arthur, 2007; Sadick et al., 2005; Iyer et al., 2018). The ability of bevacizumab to bind to and inhibit the biological activity of VEGF prevents endothelial cell proliferation and angiogenesis, and its efficacy in treating HHT-associated epistaxis has been demonstrated (Dupuis-Girod, 2020). Bevacizumab is a recombinant humanized antibody directed against vascular endothelial growth factor, and is approved by the US Food and Drug Administration for use in patients with colorectal or other cancers. After initial case reports that intravenous bevacizumab with oncology dosing improves bleeding in patients with HHT, (Flieger et al., 2006; Bose et al., 2009) subsequent reports suggest efficacy at lower doses (Suppressa et al., 2011; Thompson et al., 2014) or with submucosal24or topical treatment (Davidson et al., 2010).

The efficacy of HHT related to epistaxis in each current study was mainly assessed by duration of epistaxis, number of epistaxes and ESS. The ESS was created as a standardized measure for assessing the extent of epistaxis (Hoag et al., 2010). The ESS is based on six questions, four of which document the frequency, duration, intensity and treatment need of epistaxis, while the other two detail the presence of anemia and whether the patient requires a blood transfusion. The severity weights vary on a scale of 0–10, with 0 as disease-free and 10 for severe disease. To date, ESS has been used as an excellent measure of epistaxis severity.

Seven studies were included in this meta-analysis, dating from 2009 to 2023, from France, USA and Austria, and no significant publication bias was found by funnel mapping. The meta-analysis showed that bevacizumab reduced the ESS in HHT epistaxis patients compared with the control, but had no significant effect on the duration and number of epistaxes. In terms of adverse effects, there was no significant difference between the bevacizumab and control groups. Through sensitivity analysis, it can be seen that deleting any of the three studies on the duration, frequency, and adverse reactions of nasal bleeding between the bevacizumab group and the control group did not have a significant impact on the combined effect values of the remaining literature, confirming the stability of the results. However, in the ESS study, after deleting some of the literature, there was no statistical difference in the duration of nasal bleeding between the bevacizumab group and the control group’s ESS score, and further large-scale sampling is needed Further confirmation from multicenter clinical studies. Due to the limited number of literature included in this meta-analysis, subgroup analyses such as national and bevacizumab dosage and dosage forms cannot be conducted. The ESS score includes six influencing factors, namely: frequency, duration, intensity, need for medical care, anemia, and need for blood transfusion. From the above results, there is no significant difference in the duration and number of epistaxes, but the ESS is significantly decreased. Thus, in addition to the duration and number of epistaxes, greater attention should be paid to the two important indicators of intensity and need for treatment(such as the need for medical care, anemia, and the need for blood transfusion). However, the current literature has paid relatively little attention to these indicators, so a combined meta-analysis is not yet possible. It is hoped that in the future, a multicenter and large-sample randomized controlled trial will observe intensity and need for treatment indicators.

This study has some limitations. First, the combined sample size of the included articles was small and may have had some impact on the results. Second, the included articles were from the United States, France and Austria, which may have generated some regional bias. Third, the heterogeneity in some articles may have had a certain influence on the results of the pooled analysis. In the future, multicenter studies should be designed in order to further explore the effectiveness and safety of bevacizumab in the treatment of HHT epistaxis, thereby providing a more reliable decision basis for clinical diagnosis and treatment.

## 5 Conclusion

Bevacizumab was superior to the control group in the treatment of HHT epistaxis, and adverse reactions were not further increased in the bevacizumab group than in the control group, suggesting that bevacizumab has clinical value in the treatment of HHT epistaxis.

## Data Availability

The raw data supporting the conclusion of this article will be made available by the authors, without undue reservation.
